# Genome sequencing and population genomics modeling provide insights into the local adaptation of weeping forsythia

**DOI:** 10.1038/s41438-020-00352-7

**Published:** 2020-08-01

**Authors:** Lin-Feng Li, Samuel A. Cushman, Yan-Xia He, Yong Li

**Affiliations:** 1 Innovation Platform of Molecular Biology, College of Forestry, Henan Agricultural University, Zhengzhou, China; 2 Ministry of Education Key Laboratory for Biodiversity Science and Ecological Engineering, School of Life Sciences, Fudan University, Shanghai, 200438 China; 3 U.S. Forest Service, Rocky Mountain Research Station, 2500 S. Pine Knoll Dr., Flagstaff, Arizona USA; 4 School of Life Sciences, Henan University, Kaifeng, China

**Keywords:** Population genetics, Molecular ecology

## Abstract

Understanding the genetic basis underlying the local adaptation of nonmodel species is a fundamental goal in evolutionary biology. In this study, we explored the genetic mechanisms of the local adaptation of *Forsythia suspensa* using genome sequence and population genomics data obtained from specific-locus amplified fragment sequencing. We assembled a high-quality reference genome of weeping forsythia (Scaffold N50 = 7.3 Mb) using ultralong Nanopore reads. Then, genome-wide comparative analysis was performed for 15 natural populations of weeping forsythia across its current distribution range. Our results revealed that candidate genes associated with local adaptation are functionally correlated with solar radiation, temperature and water variables across heterogeneous environmental scenarios. In particular, solar radiation during the period of fruit development and seed drying after ripening, cold, and drought significantly contributed to the adaptive differentiation of *F. suspensa*. Natural selection exerted by environmental factors contributed substantially to the population genetic structure of *F. suspensa*. Our results supported the hypothesis that adaptive differentiation should be highly pronounced in the genes involved in signal crosstalk between different environmental variables. Our population genomics study of *F. suspensa* provides insights into the fundamental genetic mechanisms of the local adaptation of plant species to climatic gradients.

## Introduction

Spatial heterogeneity of the environment exerts differential selection pressures on natural populations, potentially leading to the local adaptation of a species across its range^1,2^. For naturally distributed plant species, selection pressure is mainly generated by abiotic factors such as soil chemistry, temperature, precipitation, and solar radiation^3^. These abiotic factors induce adaptive genetic differentiation on a genome-wide scale via spatially varying selection^4^. Adaptive divergence may lead to changes in gene expression, metabolism, physiology, phenotype, or phenology that significantly increase the adaptive ability of species across local environmental gradients^4,5^. Identifying the regions at the genome involved in local adaptation can aid in the design of appropriate protection and management strategies to reduce the impact of future climate change, in addition to providing insights into the genetic mechanism of local adaptation^6^. Genome scanning on a population scale enables the identification of genetic variants that control adaptive differences among natural populations^7,8^.

With the rapid development of sequencing technology, it is now possible to generate genomic information to understand the genomic basis underlying local adaptation^9,10^. As a result of this genomics revolution, evidence of local adaptation has greatly increased in recent decades. Some notable examples include *Apis cerana*, *Arabidopsis thaliana*, *Corymbia calophylla*, *Fundulus heteroclitus*, and *Peromyscus maniculatus*^11–15^. These empirical studies identified adaptive genetic variation on a genome-wide scale that is driven by natural selection along environmental gradients.

Different environmental factors exert different selection pressures on species, which may be due to the differential sensitivity of species to these environmental factors and how environmental gradients interact with fitness landscapes^9,16^. This raises the fundamental question of which environmental factors play key roles in promoting adaptive divergence. Recent studies have suggested that environmental factors related to ecological niche dimensions are the main forces driving adaptive differentiation^17,18^.

However, the results supporting the habitat niche selection hypothesis have been produced using low-density molecular markers, and it is doubtful whether the same conclusion will be reached when analysis is extended to genome-wide markers. An additional critical question is which genes are involved in local adaptation. Recent population genomics studies in animals and humans have shown that only a small number of genes are typically involved in local adaptation^11,14,19,20^. Compared with animals and humans, plants often exhibit low migration abilities, which may increase the strength of local selection pressures in relation to climate change and environmental heterogeneity, resulting in more adaptive genetic variations in the genome to cope with these stresses.

The recent growth of plant population genomics has provided key information and perspectives that have revolutionized research on local adaptation^15,21,22^. In some past studies of adaptive evolution, substantial effects of pleiotropy were found^23^. Moreover, many adaptive differentiation loci are frequently simultaneously associated with more than two environmental variables, which suggests that adaptive differentiation may occur more frequently in the genes showing crosstalk between different environmental modules.

Here, we propose the hypothesis that adaptive differentiation will occur at a high frequency in the genes responsible for signal crosstalk between different environmental variables during the process of local adaptation. However, most of the previous studies in this research area used molecular markers without sequence information or single-nucleotide polymorphism (SNP) markers without a reference genome^18^. These limitations make it difficult to ascertain which genes generate adaptive differentiation for local adaptation and whether adaptive differentiation occurs in genes related to signal crosstalk between different environmental factors. Conducting adaptive genomics studies on species with available reference genomic information will better answer these questions and provide a clearer understanding of the genetic mechanisms of local adaptation^4,24^.

The last question that we investigate in this paper is whether the selection pressures imposed by these environmental gradients are sufficient to affect the population genetic structure of species. In the past, we used neutral molecular markers to investigate the population genetic structure of species^25^. We focused on the effects of gene flow, genetic drift, population demographic history, and geological history^26,27^. However, natural selection generated along environmental gradients is also an important factor affecting the genetic structure. Increasing evidence of correlations between population genetic structure and environmental factors indicates a need to examine the role of natural selection in shaping the spatial structure of populations^28,29^. In addition, natural selection caused by environmental factors will also lead to the differentiation of loci around these adaptive genes due to the hitchhiking effect, which will also influence the apparent relationships between environmental factors and genetic structure. However, we do not know whether the magnification of this effect will outweigh the effects of other neutral processes, such as gene flow and drift. When molecular markers are used for analyses at the genome level, the role of natural selection in determining population genetic structure will be more readily measurable. Overall, the use of population genomics is helpful for understanding the genetic mechanisms of local species adaptation^2^.

Weeping forsythia, *F. suspensa* (Thunb.) Vahl (Oleaceae) (2*n* = 2*x* = 28), is a dominant, deciduous shrub that is widely distributed in the warm temperate zone in China^25^. In previous phylogeographical studies of *F. suspensa*, it was inferred that this species tracked quaternary climatic changes by expanding to nearby low-elevation plains in glacial periods and retreating to mountaintops during interglacial warm periods^25^. This suggests that the species has responded to environmental fluctuation through a combination of limited local range shifts, local adaptation, and phenotypic plasticity. Thus, this species may be a good candidate for studies of local adaptation given the long period of environmental selection it has experienced within a relatively fixed range compared to other species that experienced more extensive range shifts following climate fluctuations. Thus, we believe *F. suspensa* is a good model for inferring local adaptation.

A recent study on *F. suspensa* indicated that the seasonal variations in precipitation, the extreme lowest temperature, and precipitation in the wettest month play important roles in the process of population adaptive differentiation^30^. However, whether these environmental gradients are key factors explaining genomic structure still needs to be verified. Furthermore, the identity of the genes associated with the observed adaptive differentiation is still not known because of the anonymous molecular markers used in previous studies^30^. In addition, Yang et al.^30^ found that most adaptive loci (22 out of 23) are simultaneously related to multiple environmental variables, indicating substantial pleiotropy. This may indicate that most of the adaptive differentiation occurs in the genes related to crosstalk between different environmental factors.

In this study, we explored the genetic mechanisms of the local adaptation of *F. suspensa* using population genomics data from specific-locus amplified fragment sequencing (SLAF)^31^. To better understand this mechanism, we sequenced the weeping forsythia genome using Nanopore PromethION. To date, the genomes of three species in Oleaceae have been published^32–34^. The genome sequencing of *F. suspensa* will be useful for understanding the structure of Oleaceae genomes in general and the evolution of the Oleaceae family. In this study, our main objective was to address the following questions: (a) what is the genomic basis underlying local adaption? (b) can more adaptive differentiation be seen in the genes related to signal crosstalk between different environmental modules? and (c) is natural selection resulting from these environmental variables is sufficient to explain the observed population genetic structure of this species?

## Results

### Assembly and annotation of the *F. suspensa* genome

As *F. suspensa* is a nonmodel species without a reference genome, we employed both the Illumina and Nanopore platforms to perform de novo genome assembly. To estimate the genome size of *F. suspensa*, we performed preliminary sequencing using the Illumina HiSeq 2500 platform to survey the genome. A total of 64.39 Gb of data were generated on the HiSeq 2500 platform (Illumina, USA). The *K*-mer spectrum analysis suggested an estimated genome size of 701.40 Mb. With this background, we assembled the reference genome of *F. suspensa* using 113× Nanopore ultralong reads. After correction using Illumina reads, we obtained an integrated assembly of 737.5 Mb in size, which consists of a total of 1214 contigs, with a contig N50 of 7.3 Mb (Table 1). Both the BUSCO (Benchmarking Universal Single-Copy Orthologs; 91.9% of 1440 BUSCOs) and CEGMA (Core Eukaryotic Genes Mapping Approach; 97.2% of CEGs) assessments indicated high completeness of the assembled genome. We annotated 401.7 Mb (54.5% of the total length) of repetitive sequences in the *F. suspensa* genome according to repeat prediction, with the *Copia* and *Gypsy* families representing the most abundant transposable elements in the *F. suspensa* genome (Supplementary File 1: Table S1). Based on a combination of *ab initio* gene prediction, homologous identification and transcripts derived from various tissues, we annotated 33,062 protein-coding genes (Supplementary File 2: Fig. S1 and Supplementary File 1: Table S2). Approximately, 33.0–96.0% of the total annotated genes were found to be homologous to known proteins in the translated EMBL (TrEMBL) database, 96.2% in the nonredundant (NR) database, 54.7% in the eukaryotic orthologous groups (KOG) database, and 50.1% in the Kyoto Encyclopedia of Genes and Genomes (KEGG) database, and homologs were also found in other databases (Supplementary File 1: Table S3). In addition, we annotated the noncoding RNA genes in the assembly, among which 103 microRNAs, 219 ribosomal RNAs, and 681 transfer RNAs were predicted (Supplementary File 1: Table S4).

**Table 1:** Summary of the *Forsythia suspensa* genome assembly and annotation

Genome assembly	Genome size	737.47 Mb	
	GC content	33.66%	
	Contig N50	7.33 Mb	
	Longest contig	20.80 Mb	
Transposable elements	Annotation	Percent	Total length
	Retrotransposons	45.49	335.50 Mb
	DNA transposons	9.33	68.79 Mb
	Others	9.38	69.17 Mb
	Total	54.46	401.65 Mb
Protein-coding genes	Predicted genes	33,062	
	Average gene length	4259 bp	
	Average exon length	1494 bp	
	Average intron length	2765 bp	

### SNP identification and quality control

**Fig. 1:**
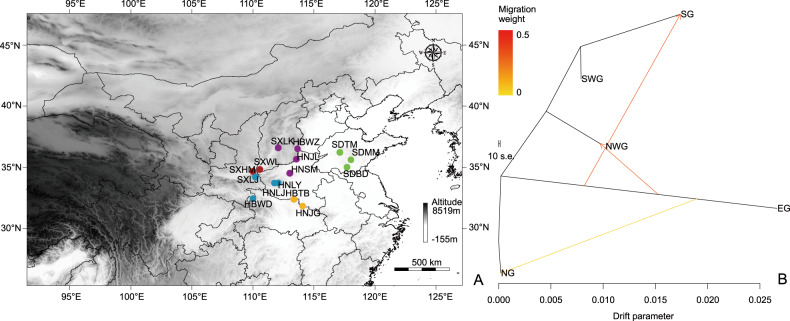
Gene flow between groups and sampling localities of *Forsythia suspensa*. **a** Sampling localities of 15 populations of *F. suspensa*, where colors distinguish groups; **b** maximum-likelihood tree and migration events among five groups of *F. suspensa*. The migration events are colored according to their weight

To provide a genome-wide overview of the genomic dynamics underlying local adaptation, a total of 300 *F. suspensa* individuals were collected from 15 natural populations across its current distribution range in China (Fig. 1a). Based on these population samples, our reduced-representation genome sequencing approach yielded 1,120,232 SLAF loci, with an average sequencing depth of 11.51× for each SLAF locus. Among these SLAF loci, 1,021,768 were polymorphic, among which 575,792 high-quality SNPs (allele frequency > 0.05 and integrity > 0.8) were used for subsequent population genetic analyses.

### Phylogenetic and population genomic analyses

**Fig. 2:**
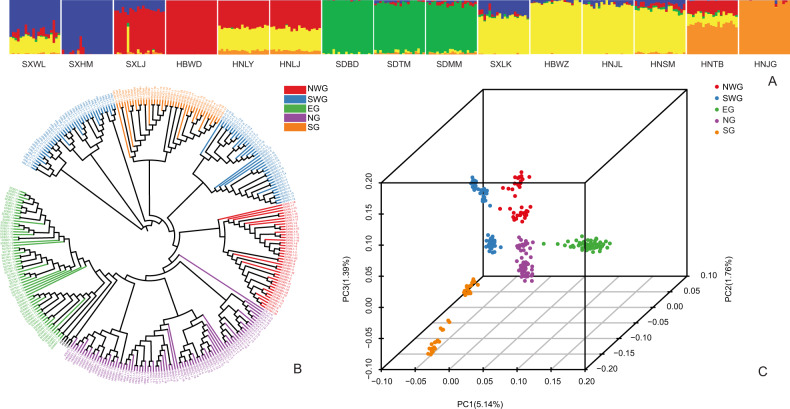
Population genetic structure of *Forsythia suspensa*. **a** Population genetic structure based on all SNPs estimated by ADMIXTURE analysis with *K* = 5. Each bar represents an individual, and the *x*-axis location specifies the sampling location. **b** The phylogenetic tree of all individuals of *F. suspensa* with *F. viridissima* and *J. nudiflorum* as outgroups was constructed using the neighbor-joining method in the program MEGA. **c** Each point represents an individual genotype, where colors distinguish groups. The amount of variation explained by PC1, PC2, and PC3 is 5.14%, 1.76% and 1.39%, respectively

The population genetic structure of *F. suspensa* was inferred using ADMIXTURE^35^ based on all high-quality SNPs. Optimal ancestral clustering at *K* = 5 was selected based on the cross-validation error rate (Supplementary File 2: Fig. S2). The resulting ancestral inference of the populations was broadly consistent with the geographic origin of the 15 populations. Thus, we defined these natural populations as the following five groups: (1) northwest group (NWG): populations SXWL and SXHM; (2) southwest group (SWG), populations SXLJ, HBWD, HNLY, and HNLJ; (3) eastern group (EG), populations SDBD, SDTM, and SDMM; (4) northern group (NG), populations SXLK, HBWZ, HNJL, and HNSM; and (5) southern group (SG), populations HNTB and HNJG (Fig. 2a).

We also reconstructed the phylogenetic relationships of the 15 populations based on the same dataset. Most results were consistent with the population structure detailed above; however, the SG group was further divided into two subgroups (Fig. 2b). Principal component analysis (PCA) also illustrated 5 distinct groups among the 15 natural populations (Fig. 2c). Notably, while all the above population genomic inferences suggested a clear spatial structure of *F. suspensa*, a relatively small proportion of the genetic variation (11.20%, *F*_ST_ = 0.112, *P* > 0.05; Table 2) could be explained by intergroup divergence. Combining the three clustering methods, PCA and ADMIXTURE based on the Bayesian algorithm showed a much clearer population structure than the phylogenetic tree. To infer the environmental correlates with the genetic structure, we performed another ADMIXTURE analysis based on the neutral loci (i.e., excluding outlier loci). The optimal clustering results of the population changed to *K* = 4 (Supplementary File 2: Fig. S3). The results of Treemix analysis showed that extensive gene flow occurred between EG and other groups, whereas there was a lack of detectable gene flow between EG and SG (Fig. 1b).

**Table 2:** AMOVAs for SNP variation surveyed in populations of *Forsythia suspensa* in China

Source of Variation	d.f.	Sum of Squares	Variance Components	Percentage of Variation (%)	*P*-value
Among groups (*F*_ST_)	4	205647.374	411.455	11.200	0.998 ± 0.010
Among individuals within groups (*F*_IS_)	295	925052.968	−125.964	−3.530	0.000 ± 0.000
Within individuals (*F*_IT_)	300	330363.000	3387.702	92.230	0.000 ± 0.000

### Characterization of candidate environment-associated loci (EAL)

**Fig. 3:**
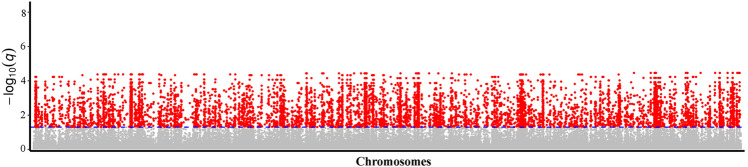
Manhattan plots showing signatures of selection in the BayeScan results. Each dot represents a SNP, and a red dot represents a SNP under selection, with a cut-off value of a posterior probability above 0.76 and *q* value below 0.05

**Fig. 4:**
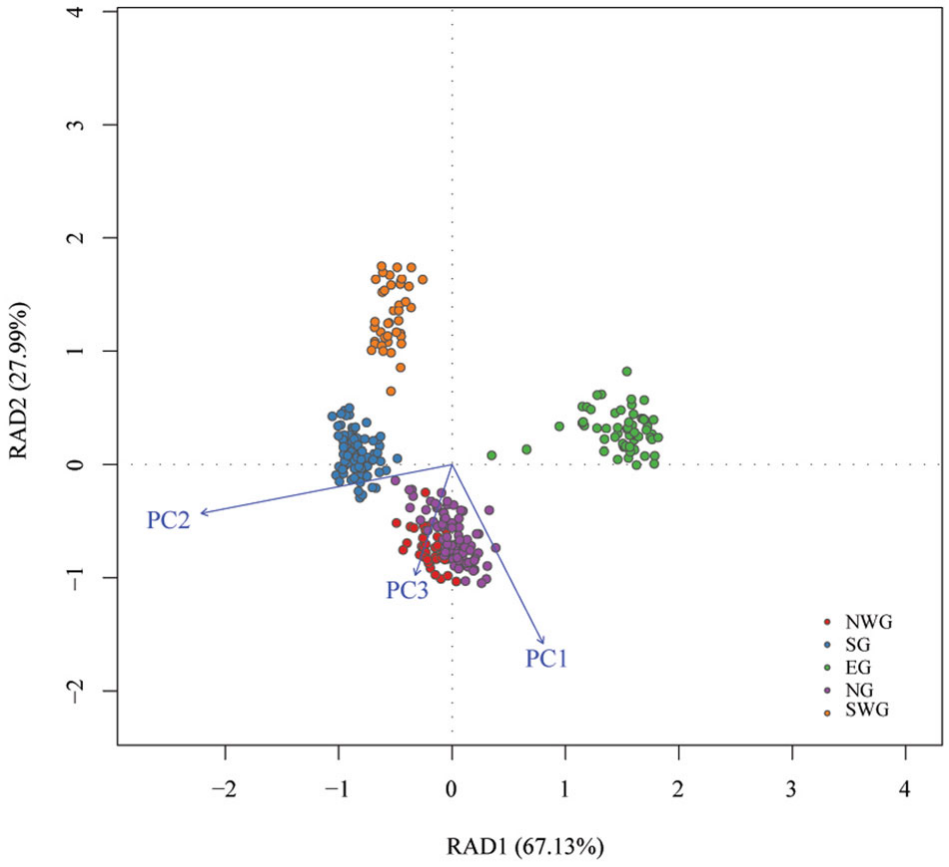
Redundancy analysis of Forsythia suspensa showing the relative contribution of each environmental variable shaping the spatial genetic structure. The biplot depicts the eigenvalues and lengths of eigenvectors for the RDA

Based on the genome-wide scan for signatures of selection, 8629 unique SNPs that possessed a posterior probability over 0.76 and *q* value under 0.05 were defined as outlier loci (Fig. 3; Supplementary File 1: Table S5). To further examine whether these loci were associated with environmental variables, both the LFMM and Samβada approaches were employed. Before the association analysis, PCA was performed to eliminate collinearity among the 43 environmental variables. Three PCs explaining 94.6% of the total variation were retained for subsequent environmental association analyses. In PC1 (56.5% of variance), the temperature variables Bio1, Bio6, Bio9, and Bio11, the precipitation variables Bio12, Bio14, Bio17, and Bio19, and the water vapor pressure variables vapr1–vapr12 showed strong negative correlations with loadings under −0.8. In PC2 (30.1% of variance), the solar radiation variables Sr8–Sr11 were strongly negatively correlated with loadings under −0.8 (Supplementary File 1: Table S6). All the above environmental variables are shown in the Supplementary material (Supplementary File 1: Table S7). As a result, a total of 722 and 6340 SNPs associated with the transformed environmental variables were identified by LFMM and Samβada, respectively (Supplementary File 2: Figs. S4 and S5; Supplementary File 1: Tables S8 and S9). To reduce the false discovery rate, only 524 mutual SNPs that were identified by both approaches were considered EAL (Supplementary File 1: Table S10). Redundancy analysis (RDA) was then performed to examine the environmental variables related to the genetic variation of these 524 candidate loci. Axes 1 and 2 of the RDA explained 67.13% and 27.99% of the variance in the 524 candidate EAL, respectively (Fig. 4). Our analyses also revealed that the PC1 and PC2 transformed environmental variables were strongly associated with RDA axes 2 and 1 (Supplementary File 1: Table S11), indicating that the two RDA axes could explain the majority of the changes in the three transformed environmental variables. PC1 represented most of the variables associated with low temperature during the cold season, the annual mean temperature, precipitation during the dry season, annual precipitation, and annual air humidity. PC2 represented solar radiation from August to November (i.e., solar radiation during the period of fruit development and seed drying after ripening). These attributes indicated that PC1 and PC2 might play important roles in the adaptive genetic differentiation of *F. suspensa*.

To further understand the functions of these identified EALs, we annotated the 524 identified SNPs using our assembled genome. A total of 1932 candidate adaptive genes were annotated by referring to our assembled genome (Supplementary File 1: Table S12).

## Discussion

Oleaceae is a medium-sized family containing approximately 600 species from 25 genera^36^. Many of these genera are economically important, such as *Fraxinus*, *Forsythia*, *Jasminum*, *Ligustrum*, *Olea*, *Osmanthus*, and *Syringa*. Currently, genomes are available for three species of the family: *F. excelsior*^32^, *O. europaea*^33^, and *O. fragrans*^34^. Here, we present a high-quality reference genome sequence of the widespread species *F. suspensa*, an important medicinal and ornamental plant. Indicating its importance as a medicinal plant, most traditional Chinese medicines for influenza treatment contain an extract of *F. suspensa* fruit^37^. It is also one of the main traditional Chinese medicines used in the recent treatment of COVID-19. This species has been widely cultivated in China in recent years. However, the varieties of this species under cultivation are very limited. The obtained genome information for *F. suspensa* will therefore be an essential resource for biological research and breeding. Here, we used genomic information in combination with a landscape genomics approach^2^ to explore the genetic basis of the local adaptation of *F. suspensa*.

Naturally distributed species experience complex, heterogeneous environments, and different environmental variables exert differential selection pressure on the associated populations^18^. When species experience selection pressures related to multiple environmental factors, the genomes of different populations will undergo complex adaptive differentiation^4^. Here, a total of 524 SNPs with signatures of natural selection along environmental gradients were identified by both LFMM and Samβada. To genomically identify these loci, we annotated them in our assembled genome. According to the standard of Fritsche et al.^38^, we searched the candidate adaptive genes in the ±100 kb SNPs of EAL. Here, we annotated a total of 1932 candidate adaptive genes.

To test the hypothesis that more adaptive differentiation will occur in the genes related to signal crosstalk between different environmental modules as a result of local adaptation to heterogeneous environmental factors, we investigated a total of 640 genes annotated by using KEGG and found multiple examples that are likely related to local adaptation (Supplementary File 1: Table S13). In our analysis, we used statistically independent composite environmental variables. PC1 represents the environmental variables related to temperature and water. We found that 286 out of 640 genes were considered candidate adaptive genes associated with PC1 based on the LFMM results. Among these genes, 87 genes were confirmed to be associated with environmental adaptation in other species and were involved in the activation or suppression of transcription, the synthesis of oxidoreductase, hormones and fatty acids, electron transport in the photosynthetic system, and other pathways.

Consistent with our hypothesis, most of these genes (56 out of 87 genes) were adaptively differentiated with respect to both temperature and water stress, suggesting that the crosstalk observed between the two modules might be a common phenomenon during the process of local adaptation. For example, APETALA2-like ethylene-responsive transcription factors (EVM0000677.1, EVM0023986.1, EVM0021041.1, and EVM0019895.1) were identified as being under divergent selection in PC1. This plant-specific transcription factor has been shown to play an important role in regulating plant growth and responses to cold, heat, salt, drought and flooding^39,40^. Peroxidase (EVM0032298.1), an enzyme that catalyzes the oxidation of a particular substrate by hydrogen peroxide, was differentially expressed under drought and low temperature stress^41,42^. Gibberellin 3-beta-dioxygenase (EVM0027506.1), one of the key enzymes involved in gibberellin synthesis, showed significant differential expression under low temperature and drought conditions^43^, and its sequence differentiation might suggest that it differentially regulates plant growth through the gibberellin pathway to adapt to different environmental conditions. 3-Ketoacyl-CoA synthase (EVM0017367.1) participates in very-long-chain fatty acid synthesis and plays a role in wax biosynthesis. A previous study of *Brassica napus* suggested that the overexpression of the genes encoding 3-ketoacyl-CoA synthase promotes cuticular wax production and increases drought tolerance^44^. Furthermore, Wang et al.^44^ found that the expression of 3-ketoacyl-CoA synthase genes was significantly downregulated by cold stress, which also indicated that they might be associated with cold adaptation. Cytochrome b6–f complex subunit 4 (EVM0014903.1), a subunit of cytochrome b6-f, plays a role in photosynthesis by transferring electrons between photosystems II and I in plants. Previous results showed the repressed accumulation of the cytochrome b6–f complex under drought stress, which suggests that it is related to drought adaptation^45^. As most of the identified divergent genes with indirect evidence in other species were simultaneously differentiated in relation to the temperature stress and water stress modules, we speculate that this might indicate that the adaptive differentiation of the genes related to crosstalk between two modules is more efficient than that of other genes. This might also be due to the climatic characteristics of this region. To verify this hypothesis, further functional verification of these divergent genes with unknown functions in temperature stress and water stress modules is still needed.

PC2 represents the solar radiation from August to November. We found that 403 out of 640 genes showed adaptive differentiation associated with PC2 based on the LFMM results. A total of 61 of these genes had been previously confirmed to be involved in the light module in other species (Supplementary File 1: Table S13). Based on previous functional annotations, we found that these divergent genes in the light module were related to light stress, photosynthesis, photoprotection and photomorphogenesis. For example, homogentisate phytyltransferase (EVM0012785.1) is a key enzyme limiting tocopherol biosynthesis. High-light stress could result in a significant elevation of total tocopherol levels and might provide an additional line of defense against oxidative damage^46^. Manganese transport protein (EVM0021805.1) is a protein located at the membrane of cells that pumps out manganese. Manganese is an essential plant mineral nutrient and plays a key role in photosynthesis, and manganese deficiency could result in a decrease in chlorophyll content^47^. Jasmonic acid-amino synthetase (EVM0011590.1) activates the conjugation between jasmonate and various amino acids, and overexpression of this enzyme causes a far-red specific hyperphotomorphogenic response^48^. The carotenoid cleavage dioxygenases (EVM0011590.1), a family of non-heme iron-containing dioxygenase enzymes, catalyze the oxidative cleavage of carotenoid substrates. Carotenoids participate in various biological processes in plants, such as photosynthesis, photomorphogenesis, photoprotection, and development^49^.

Interestingly, we found 107 candidate adaptive genes that were related to both PC1 and PC2 based on the LFMM results. According to the results of previous studies, 24 out of these 107 genes were considered candidate adaptive genes that were simultaneously involved in the adaptation to light and temperature and/or water stress modules (Supplementary File 1: Table S13). Of course, as this general inference is based on from indirect evidence from other species, we cannot completely exclude the possible existence of false-positive signals of natural selection caused by the hitchhiking effect. Thus, the candidate adaptive genes that we identified in this study require further functional verification. Importantly, however, our results strongly suggest that the adaptive differentiation of genes related to multiple metabolic pathways occurred in the genome of natural plant populations under multiple abiotic selection pressures. Our study gives us substantial insight into the genetic basis of the local adaptation of *F. suspensa*, which should form the foundation for more thorough confirmatory work in common garden experiments^9^ and simulation modeling^50^.

In previous population genomics studies of *F. suspensa*^30^, it was not possible to identify the loci responsible for adaptive differentiation due to the limitations of molecular markers. However, the key environmental factors involved in the adaptive differentiation of *F. suspensa* have been identified. Waterlogging, cold, and drought are considered to be the most important environmental factors affecting the adaptive differentiation of *F. suspensa*^30^. In this study, RDA at the genome level confirmed that cold and drought significantly contributed to the adaptive differentiation of natural populations of *F. suspensa*, but waterlogging was ruled out as a significant contributor in this analysis, although we also found some adaptive genes associated with this phenomenon, such as malate dehydrogenase (EVM0007696.1)^51^ and gibberellin 3-beta-dioxygenase (EVM0027506.1)^52^. In addition, we identified a new environmental factor that might play a key role in the adaptive differentiation of *F. suspensa*: solar radiation during the period of fruit development and seed drying after ripening, which has not been reported in previous studies.

Additional important questions are whether, when and how natural selection introduced by environmental variables will affect population genetic structure. The genetic structure of a species is affected by many factors^53,54^. The identification of population genetic structure at the genome level can help provide a comprehensive understanding of the effects of these factors on population genetic structure. Recently, three studies have described the population genetic structure of *F. suspensa*^25,30,55^. The first study of the phylogeography of *F. suspensa* was based on chloroplast DNA (cpDNA) and nuclear ribosomal DNA (nrDNA). Importantly, cpDNA-based analyses divide the population of *F. suspensa* into seven groups, whereas nrDNA cannot separate these populations at all^25^. The second study of population genetics in *F. suspensa* used microsatellite markers, in which the populations of *F. suspensa* that were not separated using nrDNA were divided into two groups, the east and west groups^57^. The third study of landscape genomics in *F. suspensa* used start codon-targeted (SCoT) polymorphism markers and further subdivided the western group identified by microsatellite markers into three groups^30^. The first two studies using nuclear molecular markers suggested that gene flow plays an important role in the population genetic structure of *F. suspensa*, while the third study using SCoT makers, a kind of nuclear gene-targeted marker, suggested that natural selection also plays an important role in shaping the population genetic structure of *F. suspensa*.

Here, our SLAF sequencing results provided 0.58 million high-quality SNPs across the genome, which enabled us to identify finer-scale population structures than in previous studies. Using these SNPs, regardless of their neutrality, our analysis provides a more comprehensive understanding of population genetic structure. Compared with phylogenetic tree methods, ADMIXTURE and PCA revealed a more refined population genetic structure of *F. suspensa*. As indicated through population genomic inferences, the 15 natural populations of *F. suspensa* could be broadly divided into 5 groups corresponding to their geographic locations. Notably, while both the PCA and ADMIXTURE inferences supported the genetic divergence of the five groups, polyphyletic relationships were observed in the NJ tree. There are three possible explanations for this phenomenon: (1) as indicated in the AMOVA, only a small proportion (11.2%) of the identified genetic variants contributed to the observed interpopulation genetic differentiation; (2) the Bayesian-based inference suggested that the *F. suspensa* populations within the same geographic group differed in their genetic constitution; and (3) historical gene flow occurred frequently among the five geographic groups. Consistent with the results of previous studies, we found that EG was separated from the other populations, and previous studies confirmed that this separation was caused by ecological isolation^25^. However, the identification of other subgroups in this study differed from the results of previous studies.

To understand the contribution of natural selection imposed by environmental variables to the population genetic structure of *F. suspensa*, we conducted a comparison between the clustering results of all SNP loci and the results excluding outlier loci. After excluding outlier loci, the east group was still separated from the other groups, whereas the clustering results of the other groups changed significantly. This further confirmed that the separation of the EG from the other populations is not caused by natural selection, but natural selection plays an important role in shaping the genetic structure of other populations of *F. suspensa*. These results are consistent with previous landscape genomics studies on *F. suspensa*^30^. Although the TREEMIX results showed gene flow between the EG and NWG, NG, and SG groups, the current amount of gene flow is not sufficient to eliminate the genetic differentiation caused by ecological isolation. In previous population genetics studies, we have paid more attention to the effects of genetic drift and gene flow on population genetic structure due to the use of neutral markers^53,54^. However, natural selection is also an important force shaping the population genetic structure of species^17^. It promotes the differentiation of adaptive genes in the genome of species among natural populations, and this genetic differentiation is further amplified due to the hitchhiking effect. This also amplifies the ability of natural selection to shape population genetic structure. Our study provides an empirical example of a species’ genetic structure being significantly influenced by natural selection imposed by multiple environmental factors.

## Conclusions

In the present study, we provide the first genome of *F. suspensa*, an important medicinal and ornamental plant. The genome information of *F. suspensa* is an essential resource for biological research and breeding in this species. We used this genome information in combination with a landscape genomics approach to explore the genetic basis of the local adaptation of this species. Our results showed that the genes of *F. suspensa* in the light, temperature and water modules have undergone adaptive differentiation under the effects of heterogeneous environmental gradients. We also found extensive signal crosstalk involving these adaptive genes between different modules. Among these environmental factors, solar radiation during the period of fruit development and seed drying after ripening, cold, and drought were found to significantly contribute to the adaptive differentiation of *F. suspensa*. Natural selection driven by multiple environmental factors has significantly contributed to the population genetic structure of *F. suspensa*. Overall, our study provides insights into the genetic mechanisms of local adaptation in this species.

## Materials and methods

### Sampling

The specimen used for de novo assembly was collected from the Zhengzhou Botanical Garden (voucher no. 2018LIFS021). The 300 *F. suspensa* samples were collected from 15 geographically isolated locations (voucher No. 2018LIFS001–2018LIFS015) covering its current distribution range in China (Fig. 1a; Table 3). In addition, two samples of *Forsythiaviridissima* and *Jasminum nudiflorum* were collected from Henan Agricultural University. The population samples used for SLAF sequencing were placed in silica gel at room temperature after collection. All samples included in this study were used according to Chinese regulations. All voucher specimens were identified by Dr. Yong Li.

**Table 3:** Details of the population locations of *Forsythia suspensa* sampled in China

Population no. and code	Locations	Lat.(N)/Long.(E)	*N*
*Northwest group*			
1. SXWL	Wulaofeng, Shanxi	34.81/110.58	20
2. SXHM	Hua Mt., Shaanxi	34.52/110.08	20
*Southwest group*			
3. SXLJ	Laojun Mt., Shaanxi	34.33/110.20	20
4. HBWD	Wudang Mt., Hubei	32.42/110.01	20
5. HNLY	Longyuwan, Henan	33.67/111.79	20
6. HNLJ	Laojieling, Henan	33.66/111.77	20
*Eastern group*			
7. SDBD	Baodugu, Shandong	34.99/117.71	20
8. SDTM	Tai Mt., Shandong	36.21/117.12	20
9. SDMM	Meng Mt., Shandong	35.56/117.97	20
*Northern group*			
10. SXLK	Lingkong Mt., Shanxi	36.59/112.08	20
11. HBWZ	Wuzhi Mt., Hebei	36.51/113.65	20
12. HNJL	Jiulian Mt., Henan	35.64/113.55	20
13. HNSM	Song Mt., Henan	34.50/113.02	20
*Southern group*			
14. HNTB	Tongbai Mt., Henan	32.36/113.36	20
15. HNJG	Jigong Mt., Henan	31.80/114.08	20

*N* number of individuals

### Genome sequencing and assembly, gene and repeat annotation

Genomic DNA was extracted from fresh young leaves by using plant DNA extraction kits (Tiangen, Beijing, China). DNA was quantified using a NanoDrop One microvolume UV–vis spectrophotometer (Thermo Fisher, Wilmington, USA). The genome size of *F. suspensa* was estimated based on the Illumina short insert library (350 bp) using Jellyfish^56^. The library for Nanopore PromethION sequencing was prepared according the protocol of Jain et al.^57^. The concentration of the DNA library was assessed with a Qubit fluorometer (Thermo Fisher). After the construction of the library, the sample was added into the flow cell, and the flow cell was transferred to a Nanopore PromethION sequencer at Biomarker Technologies (Beijing, China) for real-time single-molecule sequencing to obtain original sequencing data. The read quality values of the original data sequenced on the Nanopore sequencing platform were preliminarily filtered to remove reads with low quality and a length of less than 2 kb. Reads after preliminary filtration were corrected and assembled using the program Canu^58^. Then, WTDBG2 and SMARTdenovo were used to assemble the error-corrected data from Canu. Quickmerge^59^ was used to integrate the assembly results yielded by WTDBG2 and SMARTdenovo, and the integration results were corrected with Racon software^60^. To improve the accuracy of the genome sequences of *F. suspensa*, we used the Illumina data to further calibrate the sequencing data of Nanopore PromethION. After correction with Pilon software^61^, the final version of the genome of *F. suspensa* was obtained. The genome completeness was assessed using a terrestrial plant dataset from BUSCO version 2.0^62^ and a eukaryote dataset from CEGMA version 2.5^63^. The protocol for gene and repeat annotation is provided in Supplementary File 2: Protocol S1.

### Genotyping by SLAF sequencing

The genomic DNA of the 300 *F. suspensa* and four outgroup samples was extracted from the silica gel-dried leaf material using a Plant DNA Extraction Kit (Tiangen, Beijing, China). DNA was quantified with Nanodrop One UV–vis spectrophotometers (Thermo Fisher Scientific, Waltham, MA, USA). In this experiment, an improved SLAF sequencing strategy was used to construct the sequencing library. Using our own assembled genome as a reference for electron digestion prediction, two enzymes (HaeIII-Hpy166II, New England Biolabs, Ipswich, MA, USA) were selected for the digestion of genomic DNA. The SLAF-seq library was constructed using a customized version of Zhang et al.’s^64^ protocol. Libraries were sequenced using 125-bp paired end reads on the Illumina HiSeq 2500 platform (Illumina, Inc., San Diego, CA, USA) at Biomarker Technologies (Beijing, China). The low-quality reads (quality score < 30e) were removed, and the rest of the reads were sorted for each sample according to duplex barcode sequences. Clean reads from the same sample were mapped onto the genome sequence of *F. suspensa* using BWA software^65^. Sequences mapping to the same position with over 95% identity were considered to represent a single SLAF locus. Using GATK^66^ and SAMtools^67^ to develop SNP markers, the intersection of the SNPs obtained via the two methods was considered to represent the final reliable dataset. After filtering out the allele tags of each SLAF locus with a minor allele frequency under 5% and integrity under 80%, the remaining high-quality SNPs were screened for subsequent population genetic analyses.

### Population genetic analysis

Population genomic analyses of the collected samples were performed based on PCA, admixture analysis, and phylogenetic inferences. PCA was performed by using EIGENSOFT version 6.0^68^. To investigate the maximum likelihood of the ancestry of each individual, population structure was inferred by using ADMIXTURE version 1.2^34^ with a range of 1–10 coancestry clusters. The optimal *K* value was determined according to the valley value of the error rate. The genetic relationships of all samples of *F. suspensa* and the four outgroup samples were constructed using the neighbor-joining method in the program MEGA version 10.0^69^. The running parameters were set as follows: Kimura 2-parameter model with 1000 bootstrap replicates. According to the grouping information of ADMIXTURE, Arlequin version 3.5^70^ was used to calculate the variation between individuals within and among all groups. Treemix version 1.1^71^ was used to infer population isolation and mixing patterns, and the direction and degree of gene flow were estimated based on the frequency variation of SNPs between different groups.

### Environmental association analysis and functional annotation

To identify the candidate environmental association loci (EAL), we identified outlier loci that were assumed under natural selection and detected them using BayeScan version 2.1^72^, with the following parameters: sample size of 5000, thinning interval of 10, 20 pilot runs with a 5000 run length, 50,000 burn-in iterations, and 10 prior odds. The cut-off thresholds were a posterior probability over 0.76 and a *q* value lower than 0.05, which are considered to provide substantial evidence of selection. Then, a total of 43 environmental variables (Supplementary File 1: Tables S8 and S14) recorded from 1970 to 2000 at a 2.5 arcmin resolution were used in this study, including 11 temperature variables, 8 precipitation variables, 12 solar radiation variables, and 12 water vapor pressure variables. All environmental variables were downloaded from Worldclim (http://www.diva-gis.org/climate) and extracted using DIVA-GIS 7.5^73^. To eliminate the collinearity of environmental variables, PCA was performed using the FactoMineR package in R. The original environmental data were standardized with the SCALE function prior to PCA. The first method was a latent factor mixed model (LFMM)^74^, which was run with the R script of LFMM version 1.3. The running parameters were 100,000 sweeps and 50,000 burn-in sweeps. The number of latent factors was the optimal *K* value from the results of Admixture based on the neutral loci. The loci with |*z*| values over 4 and *P* values lower than 0.001 were identified as EAL. The second method was a logistic regression model run in Samβada version 0.8^75,76^. The cut-off value was set as a *P* value of the *G* score lower than 0.01 after Bonferroni correction. To infer the influence of these transformed environmental variables on adaptive genetic differentiation, RDA was performed using the vegan package^77^ in R. Here, the SNPs of EAL were used as response variables (Supplementary File 1: Table S10), and the transformed environmental variables (the PC axes) (Supplementary File 1: Table S15) were used as explanatory variables. For functional annotation, the candidate SNPs of EAL were annotated with our assembled genome of *F. suspensa* using the program BLAST X^78^ with an *E* value ≤ 10^−5^. To avoid missing candidate genes due to hitchhiking effects, the size of the sliding window for gene annotation was set as ±100 kb for SNPs of EAL based on the standard of Fritsche et al.^38^.

## Supplementary information

Supplementary Information

Supplementary Information

## Data Availability

Sequence data are archived at the National Center for Biotechnology Information (Information of SLAF sequencing: accession Nos. SRR10064062–SRR10064365, BioProject PRJNA563244; genome sequence information: accession No. WIPI00000000, BioProject PRJNA562106).
