# Supplementary material for: Genome sequencing and population genomics modeling provide insights into the local adaptation of weeping forsythia
Source: Hortic Res. 2020 Aug 1;7:130. doi: 10.1038/s41438-020-00352-7 (PMC7395120; doi:10.1038/s41438-020-00352-7)
Supplement: Supplementary file 1 — Supplementary Information [file 41438_2020_352_MOESM1_ESM.doc]

**Supplementary Protocol S1** Gene and repeat annotation.

Because of the relatively low conservatism of inter-species repeat sequences, it is necessary to construct a specific repeat sequence database when predicting repeat sequences for specific species. Therefore, we built a genome repeat sequence database based on the principle of structural prediction and *ab* *initio* prediction using the programs LTR Finder version 1.05 ^1^, RepeatScout version 1.0.5 ^2^, Piler-DF version 2.4 ^3^. The database was classified by PASTEClassifier ^4^, and then merged using Repbase ^5^. Then RepeatMasker ^6^ was used to predict the repeat sequence based on the established repeat sequence database.

To predict protein-coding genes, the three approaches were used as follows: *Ab initio* method, homology-based method, and RNAseq-based method. For *Ab initio* method, Genscan 7, Augustus version 2.4 ^8^, GlimmerHMM version 3.0.4 ^9^, GeneID version 1.4 ^10^, and SNAP ^11^ were used. For the homology-based method, GeMoMa version 1.3.1 ^12^ was used for predictions based on homologous species, *Erythranthe guttata*, *Olea europaea*, and *Sesamum indicum.* In RNAseq-based method, RNA-Seq data from bud, leaf, root and stem were generated from the Illumina platform. Then, Hisat version 2.0.4 and Stringtie version 1.2.3 ^13^ were used for the annotation with the genome as reference, and TransDecoder version 2.0 ^14^ and GeneMarkS-T version 5.1 ^15^ were used for gene prediction. PASA version 2.0.2 ^16^ was used to predict unigenes using the assembly data without reference. Finally, EVM version 1.1.1 ^17^ was used to integrate the prediction results obtained by the above three methods. The original transcriptome data were compared with the genome using TopHat ^18^, and the number of bases in the exon, intron and intergenic regions were counted for the evaluation of the gene prediction results.

Non-coding RNAs are those that do not encode proteins, including microRNA, rRNA, tRNA and other RNAs. According to the structural characteristics of different non-coding RNAs, different strategies are adopted to predict different non-coding RNAs. rRNA and microRNA were predicted using Infenal version 1.1 ^19^ based on Rfam ^20^ and miRBase ^21^ databases, and Trnascan-SE version 1.3.1 ^22^ was used to identify tRNA.

Pseudogenes are those sequences similar to that of functional genes, whereas which lose their original functions due to mutations such as insertion or deletion. Through comparison of GenBlastA version 1.0.4 ^23^, homologous gene sequences (possible genes) were searched on the genome after shielding the true gene loci, and then GeneWise version 2.4.1 ^24^ was used to search for immature termination codon and code shift mutations in the gene sequences to obtain pseudogenes.

The predicted gene sequences were compared with NR, KOG ^25^, GO ^26^, KEGG ^27^, and TrEMBL ^28^ databases using BLAST version 2.2 ^29^ with the cutoff value E-value of 1e-5 for functional annotation analysis.

**References**

1. Xu, Z. & Wang, H. LTR_FINDER: an efficient tool for the prediction of full-length LTR retrotransposons. *Nucleic Acids Res* **35**, W265–W268 (2007).
2. Price, A. L., Jones, N. C. & Pevzner, P. A. De novo identification of repeat families in large genomes. *Bioinformatics* **21**, i351–i358 (2005).
3. Edgar, R. C. & Myers, E. W. PILER: identification and classification of genomic repeats. *Bioinformatics* **21**, i152–i158 (2005).
4. Wicker, T. et al. A unified classification system for eukaryotic transposable elements. *Nat Rev Genet* 8, 973–982 (2007).
5. Jurka, J. et al. Repbase Update, a database of eukaryotic repetitive elements. *Cytogenet Genome Res* **110**, 462–467 (2005).
6. Tarailo-Graovac, M. & Chen, N. Using RepeatMasker to identify repetitive elements in genomic sequences. *Curr Protoc Bioinformatics* **25**, 4.10.1–4.10. 14 (2009).
7. Burge, C. & Karlin, S. Prediction of complete gene structures in human genomic DNA. *J Mol Biol* **268**, 78–94 (1997).
8. Stanke, M. & Waack, S. Gene prediction with a hidden Markov model and a new intron submodel. *Bioinformatics* **19**, ii215–ii225 (2003).
9. Majoros, W. H., Pertea, M. & Salzberg, S. L. TigrScan and GlimmerHMM: two open source *ab initio* eukaryotic gene-finders. *Bioinformatics* 20, 2878–2879 (2004).
10. Blanco, E., Parra, G. & Guigó, R. Using geneid to identify genes. *Curr Protoc Bioinformatics* **18**, 4.3.1–4.3.28 (2007).
11. Korf, I. Gene finding in novel genomes. *BMC bioinformatics* **5**, 59 (2004).
12. Keilwagen, J. et al. Using intron position conservation for homology-based gene prediction. *Nucleic Acids Res* 44, e89 (2016).
13. Pertea, M. et al. Transcript-level expression analysis of RNA-seq experiments with HISAT, StringTie and Ballgown. *Nat Protoc* **11**, 1650–1667 (2016).
14. Haas, B. J. & Papanicolaou, A. TransDecoder. https:github.com/TransDecoder/TransDecoder/wiki (2016).
15. Tang, S., Lomsadze, A. & Borodovsky, M. Identification of protein coding regions in RNA transcripts. *Nucleic Acids Res* **43**, e78 (2015).
16. Haas, B. J. et al. Improving the *Arabidopsis* genome annotation using maximal transcript alignment assemblies. *Nucleic Acids Res* **31**, 5654–5666 (2003).
17. Haas, B. J. et al. Automated eukaryotic gene structure annotation using EVidenceModeler and the Program to Assemble Spliced Alignments. *Genome Biol* **9**, R7 (2008).
18. Trapnell, C. Pachter, L. & Salzberg, S. L. TopHat: discovering splice junctions with RNA-Seq. *Bioinformatics* **25**, 1105–1111 (2009).
19. Nawrocki, E. P., & Eddy, S. R. Infernal 1.1: 100-fold faster RNA homology searches. *Bioinformatics* **29**, 2933–2935 (2013).
20. Griffiths-Jones, S. et al. Rfam: annotating non-coding RNAs in complete genomes. *Nucleic Acids Res* **33**, D121–D124 (2005).
21. Griffiths-Jones, S. et al. miRBase: microRNA sequences, targets and gene nomenclature. *Nucleic Acids Res* **34**, D140–D144 (2006).
22. Lowe, T. M. & Eddy, S. R. tRNAscan-SE: a program for improved detection of transfer RNA genes in genomic sequence. *Nucleic Acids Res* **25**, 955–964 (1997).
23. She, R. et al. genBlastA: enabling BLAST to identify homologous gene sequences. *Genome Res* **19**, 143–149 (2009).
24. Birney, E., Clamp, M. & Durbin, R. GeneWise and genomewise. *Genome Res* **14**, 988–995 (2004).
25. Tatusov, R. L. et al. The COG database: new developments in phylogenetic classification of proteins from complete genomes. *Nucleic Acids Res* **29**, 22–28 (2001).
26. Dimmer, E. C. et al. The UniProt-GO Annotation database in 2011. *Nucleic Acids Res* **40**, D565–D570 (2012).
27. Kanehisaa, M. & Goto, S. KEGG: kyoto encyclopedia of genes and genomes. *Nucleic Acids Res* **28**, 27–30 (2000).
28. Boeckmann, B. et al. The SWISS-PROT protein knowledgebase and its supplement TrEMBL in 2003. *Nucleic Acids Res* **31**, 365–370 (2003).
29. Altschul, S. F. et al. Basic local alignment search tool. *J Mol Biol* **215**, 403–410 (1990).

**Supplementary Figures**

**
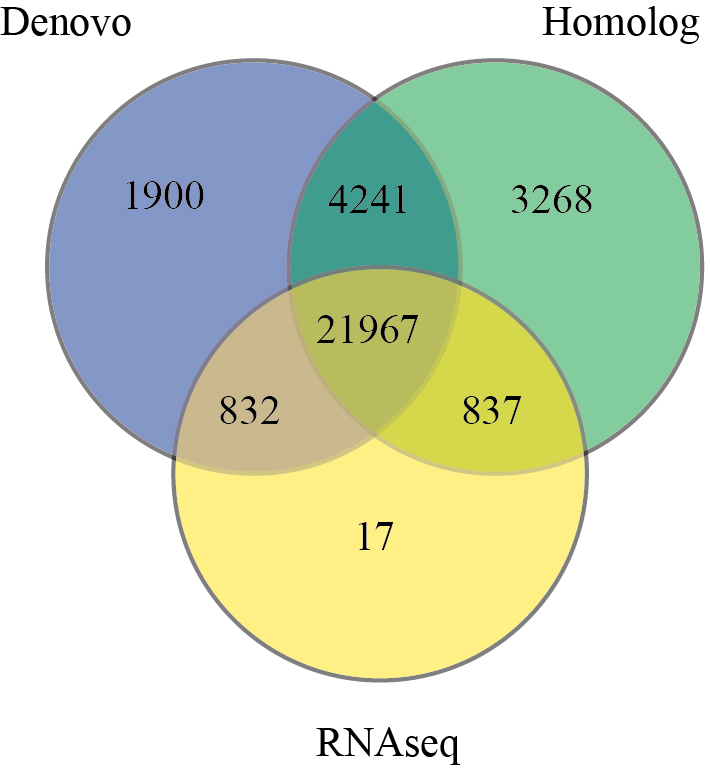
**

**Figure S1.** Distribution maps of integrated genes derived from three prediction modes.

**
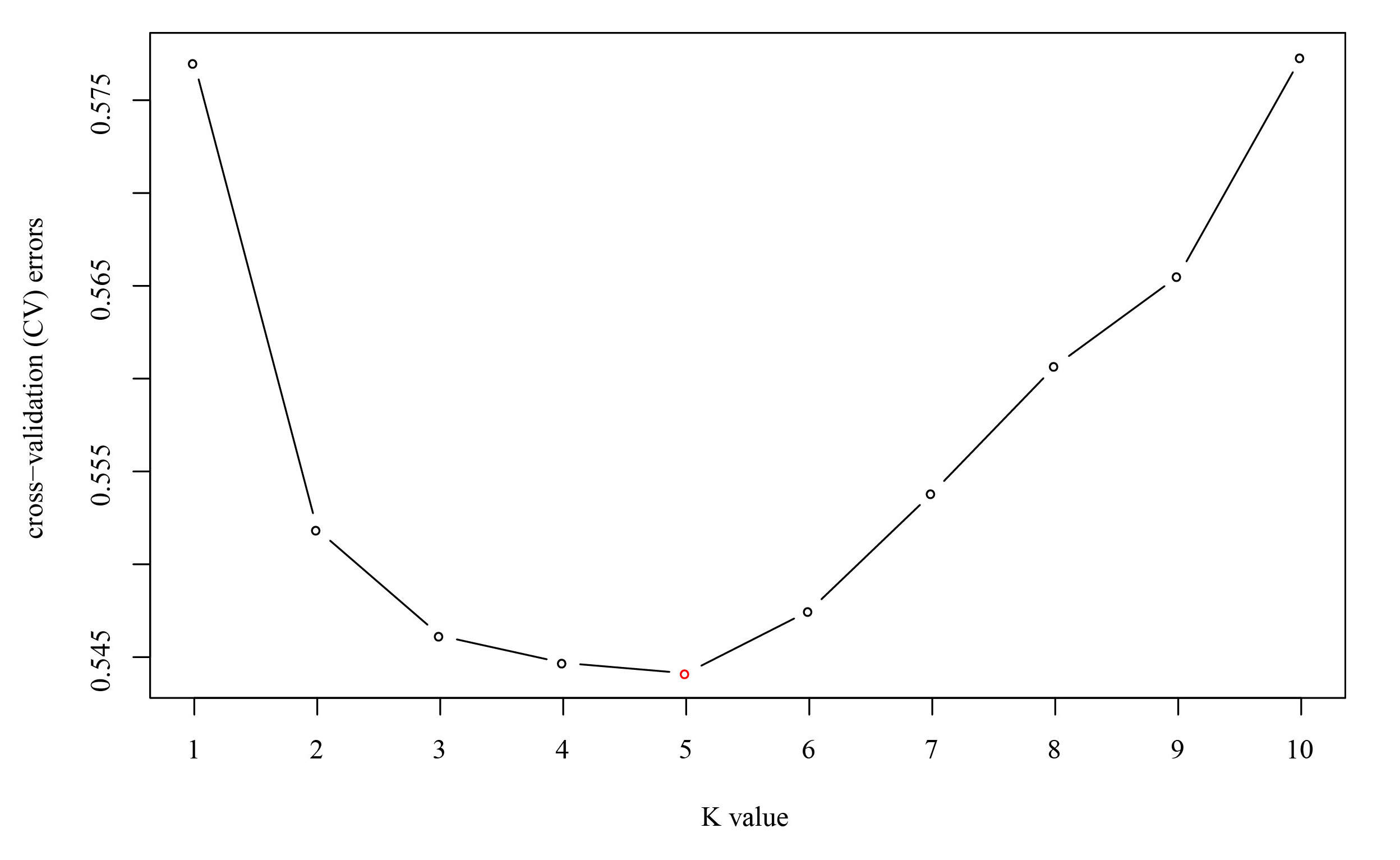
**

**Figure S2.** The optimal clustering at *K* = 5 groups were supported based on the cross-validation error rate.


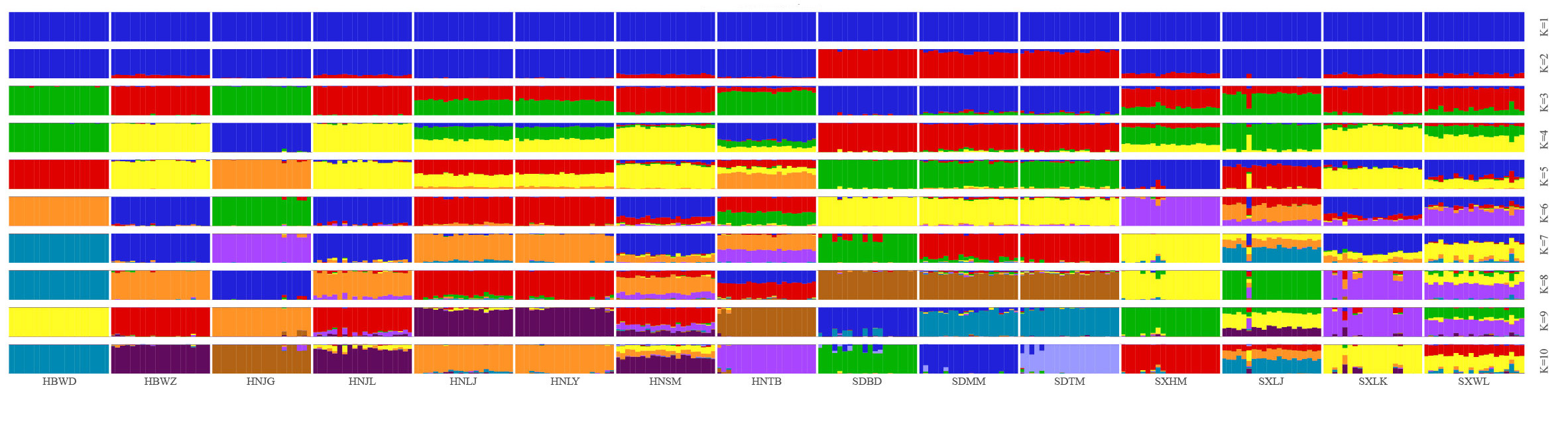


**Figure S3.** Population genetic structure based on neutral SNPs estimated by ADMIXTURE analysis with *K* = 1 to 10. Each bar represents an individual and x-axis location specifies sampling location.

##
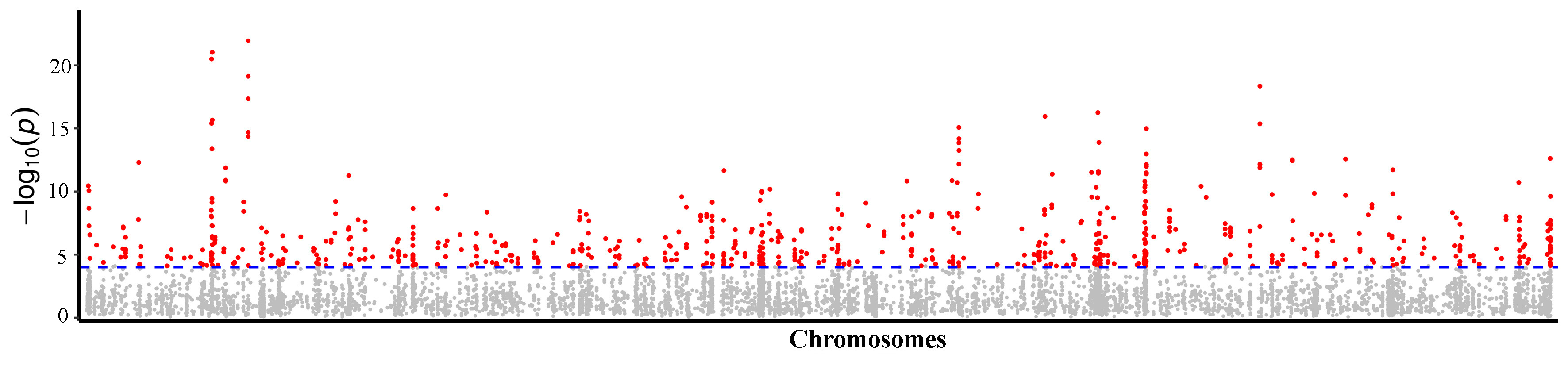


**Figure S4.** Manhattan plots showing a signatures of environment-associated loci of the results of LFMM. Each dot represents a SNP, red dot represents a SNP under selection with the cut off value of with |z| value over 4 and P-value lower than 0.001.


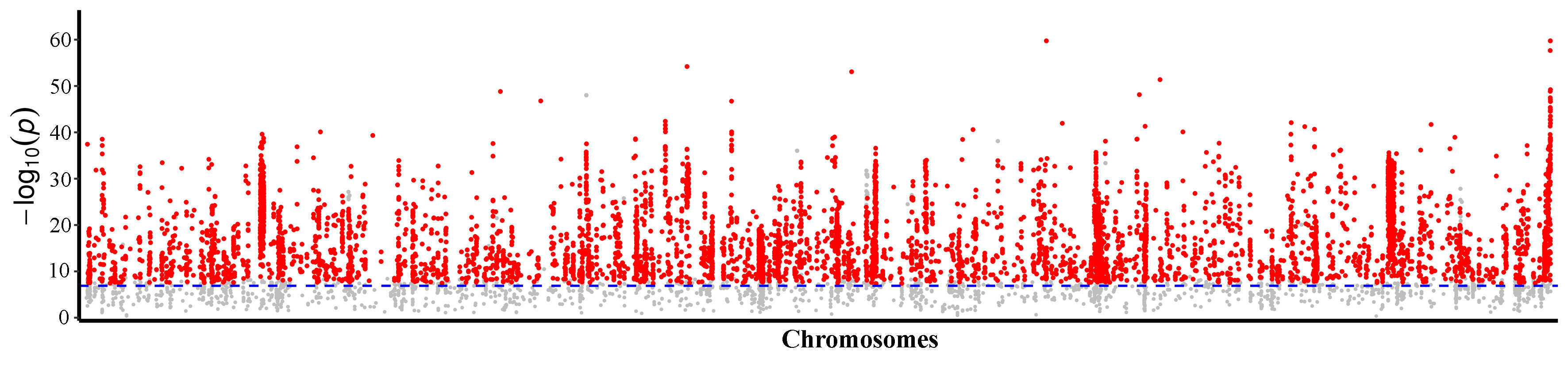


**Figure S5.** Manhattan plots showing a signatures of environment-associated loci of the results of Samβada. Each dot represents a SNP, red dot represents a SNP under selection with the cut off value of with P-value of G score lower than 0.01.

Overview List of Supplementary Tables (see the excel files)

**Supplementary Table S1.** Statistical information of repeated sequences.

**Supplementary Table S2.** Statistics of gene prediction results.

**Supplementary Table S3.** Statistical results of non-coding RNA.

**Supplementary Table S4.** Statistical information of gene function annotation.

**Supplementary Table S5.** Outlier loci identified by BayeScan.

**Supplementary Table S6.** Correlations between envirmental variables and PC axes.

**Supplementary Table S7.** Fourty-three environmental variables used in this study.

**Supplementary Table S8.** The EAL identified by LFMM.

**Supplementary Table S9.** The EAL identified by Samβada.

**Supplementary Table S10.** The EAL identified by both LFMM and Samβada.

**Supplementary Table S11.** Correlations between PC axes and RDA axes.

**Supplementary Table S12.** Annotation for SNPs identified by both LFMM and Samβada.

**Supplementary Table S13.** The adative genes assiociated with PC axes.

**Supplementary Table S14.** The envirmental variables used in this study.

**Supplementary Table S15.** The transformed environment variables of PC axes.
